# Circulating microRNAs as Prognostic and Predictive Biomarkers in Patients with Colorectal Cancer

**DOI:** 10.3390/ncrna2020005

**Published:** 2016-06-01

**Authors:** Jakob Vasehus Schou, Julia Sidenius Johansen, Dorte Nielsen, Simona Rossi

**Affiliations:** 1 Department of Oncology, Herlev & Gentofte Hospital, DK-2730 Herlev, Denmark; jakob.hagen.vasehus.schou@regionh.dk (J.V.S.); 2 Institute of Clinical Medicine, Faculty of Health and Medical Sciences, University of Copenhagen, Nørregade 10, DK-1165 Copenhagen, Denmark; julia.johansen@post3.tele.dk (J.S.J.); Dorte.Nielsen.01@regionh.dk (D.N.); 3 Institute of Oncology Research (IOR), Oncology Institute of Southern Switzerland (IOSI), Via Vela 6, CH-6500 Bellinzona, Switzerland

**Keywords:** circulating microRNA, colorectal cancer, prognostic, predictive, review

## Abstract

MiRNAs are suggested as promising cancer biomarkers. They are stable and extractable from a variety of clinical tissue specimens (fresh frozen or formalin fixed paraffin embedded tissue) and a variety of body fluids (e.g., blood, urine, saliva). However, there are several challenges that need to be solved, considering their potential as biomarkers in cancer, such as lack of consistency between biomarker panels in independent studies due to lack of standardized sample handling and processing, use of inconsistent normalization approaches, and differences in patients populations. Focusing on colorectal cancer (CRC), divergent results regarding circulating miRNAs as prognostic or predictive biomarkers are reported in the literature. In the present review, we summarize the current data on circulating miRNAs as prognostic/predictive biomarkers in patients with localized and metastatic CRC (mCRC).

## 1. Introduction

Colorectal cancer (CRC) is one of the most frequent causes of cancer-related death [1]. Early diagnosis and treatment of patients with CRC have improved over the last decade, but research in circulating biomarkers aiming to allocate patients into different prognostic groups and to predict treatment outcome is sparse.

MicroRNAs (miRNAs) are small and single-stranded nucleotides (approximately 22 nucleotides long). They are non-coding and regulate gene expression at a posttranscriptional level [2]. Many miRNAs are deregulated in cancer and can function as oncogenes or tumor suppressors [3]. A close resemblance in miRNA expression between primary CRC tumor and corresponding metastasis was recently reported [4]. Circulating miRNAs as biomarkers in patients with cancers were originally reported in serum of patients with large B-cell lymphoma [5] and plasma of patients with prostate cancer [6]. MiRNAs are transcribed intracellularly, but circulating miRNAs may enter the bloodstream through passive leakage due to tissue damage and cell apoptosis, by secretion through microvesicles and exosomes, or bound to proteins like HDL, LDL, or AGO2 [7,8,9,10].

Collection of circulating miRNAs is a non-invasive procedure and miRNAs are stable in whole blood, serum, and plasma. This is convenient for the patient, and the biomarker sampling can be repeated throughout a treatment period.

In frequently reported studies, circulating miRNAs are used as diagnostic signatures to distinguish between healthy subjects and patients with cancer. Diagnostic miRNAs can potentially be used for early detection of cancer. A prognostic miRNA profile can provide information of disease outcome independent of any given treatment given, compared to a predictive miRNA profile which provides information on a treatment effect. 

Various steps in the miRNA analyses can affect the results. In the pre-analytical setting, the choice of collection tubes, blood cell count, hemolysis, and volume of the collected sample may influence the results [11]. Important issues in the analytical and post-analytical steps are the choice of platform, normalization method, and statistical considerations such as population size, use of validation cohort, and significance threshold [12].

We conducted a review of the existing literature on the use of circulating miRNAs as prognostic or predictive biomarkers in patients with CRC. The studies included were found with a search strategy in PubMed using the terms: “colorectal cancer” AND (microRNA or miRNA) AND (blood OR circulating OR serum OR plasma OR cell free OR non-invasive). From 194 retrieved papers, we included only peer-reviewed human studies in English and papers reporting on prognostic or predictive circulating miRNAs published before 1 May 2015. Studies regarding diagnostic miRNAs were only included if the authors had prognostic or predictive data as secondary endpoints. 

We did not discard any studies due to a low number of patients included, lack of validation, or publication date. All stages of CRC were accepted. After this filtering, 17 studies were included in the review.

## 2. Results

### 2.1. Results from Studies with miR-21 as a Circulating Biomarker

We conducted a review of existing results on circulating miRNAs as prognostic and/or predictive markers. The included studies are summarized in Table 1 and grouped into circulating miR-21, solely prognostic circulating miRNAs, and miRNAs that are potentially predictive for a specific treatment.

miR-21 is an oncogenic miRNA and tissue miR-21 is correlated with poor overall survival (OS) in CRC patients [13,14,15]. Many studies have focused on this miRNA in tissue samples, and few have adapted the hypotheses that miR-21 might be important as a circulating biomarker in the diagnostic and prognostic setting. 

In a study from 2013, serum levels of miR-21 and miR-31 were tested in a small discovery population of 12 patients with CRC. Only miR-21 was validated in an independent cohort of 186 patients. Patients with high miR-21 expression were associated with short OS when divided into two groups with high and low expression. Univariate Cox analysis showed correlation with high miR-21 levels and lymph node metastases and distant recurrences. In the multivariable analysis, a high level of miR-21 in serum, but not in tissue, was an independent prognostic factor for OS [16]. 

Liu and colleagues analyzed serum levels of miR-18a, miR-21, miR-31, miR-92a, and miR-106a in a diagnostic setting of 200 patients with CRC. Among prognostic miRNAs, only high expression of miR-92a was significantly and independently associated with short OS. Circulating miR-21 was not found to be a prognostic factor in this study [17]. 

Conversely, a low expression of serum miR-21 was associated with poor clinical outcome in a study that included 102 patients with a wide range of stages and treatments. In a multivariable Cox regression analysis, low miR-21 was, together with high age, tumor stage and serum CEA, an independent prognostic factor for short OS along with CEA, age, and tumor stage [18].

In 103 patients with all stages of CRC, plasma levels of miR-21, miR-221, and miR-222 were obtained before surgery. In addition, p53, CEA, estrogen-receptor status, and progesterone-receptor status were determined by immunohistochemistry and correlated with the miRNA findings. In a multivariable analysis, elevated plasma miR-221 was correlated with poor OS along with clinical stage and histological grade. The plasma level of miR-221 was correlated with p53 expression in formalin-fixed and paraffin-embedded tissues (FFPE). MiR-21 and miR-22 were not reported as prognostic in this study [19].

Finally, Monzo *et al.* measured expression of miR-21 in plasma from peripheral veins and mesenteric veins in 57 patients during surgery of their primary CRC. In plasma from mesenteric veins, miR-21 expression was higher than in plasma from peripheral veins. High plasma levels of miR-21 in blood samples from both mesenteric and peripheral veins were associated with short disease-free survival. No validation was performed, and different patient classifications were used [20]. 

### 2.2. Results from Studies with Circulating miRNAs as Prognostic for Outcome in CRC Patients

The majority of the studies from our PubMed search investigated the diagnostic effect of circulating miRNAs as their primary endpoint. From the 194 studies screened for our review, analyses on supplementary prognostic data were found in nine of them. In eight additional studies, circulating miRNAs as prognostic or predictive were analyzed as primary endpoints. 

Toiyama *et al.* investigated five miRNAs from the miR-200 family. Members of the miR-200 family have been suggested to be important initiators of the epithelial-to-mesenchymal transition (EMT) and potentially play a role in CRC. Based on this assumption, the authors investigated the serum expression of miR-141, miR-200a, miR-200b, miR-200c, and miR-429. These miRNAs were first tested in 12 patients with stages I-IV CRC and validated in 182 CRC patients. A high serum expression of miR-200c was correlated with lymph node metastases, recurrent disease, and distant metastases. In addition, high miR-200c was an independent prognostic factor for short OS in all stages of CRC [21].

In a study by Cheng *et al.* plasma levels of miR-141 were associated with stage IV CRC and poor OS. Plasma samples were obtained from 102 patients with all stages of CRC and subsequently validated in a matched cohort of 156 CRC patients [22].

Serum samples were analyzed from 113 patients with localized or metastatic CRC (30 patients for training and the remaining 83 for validation). Samples were taken prior to surgery, chemotherapy, or radiotherapy. The primary objectives were to test the diagnostic significance of miR-21, let-7g, miR-31, miR-92a, miR-181b, and miR-203. As an extra finding, these six miRNAs were correlated with tumor stage [23].

In 146 patients (the majority with localized CRC and fewer with metastatic disease) who underwent surgery, Lv and co-workers analyzed serum levels of miR-155. High expression of serum miR-155 was correlated with shorter OS and progression free survival (PFS) in all 146 patients, but patients with stage I had lower expression than did patients with stages II–IV [24]. 

From a study by Jinushi *et al.*, interactions between miRNAs and the enhancer of zeste homolog 2 (EZH2) gene and milk fat globule-epidermal growth factor 8 (MFGE8) gene were assessed. EZH2 is associated with histone methyltransferase activity, and its expression is correlated with tumor aggressiveness, metastasis, and poor prognosis. MFGE8 is reported to be involved in tumor progression and prognosis. A group of miRNAs, potentially targeting EZH2 and MFGE8, were chosen for analysis. Seventy-one patients with both resectable and unresectable CRC treated with chemotherapy were included. High miR-124-5p and low miR-26a were correlated with prolonged OS when expression levels were dichotomized into above and below the median level [25]. 

### 2.3. Results from Studies with Circulating miRNAs as Potentially Predictive Biomarkers in Treatment of CRC

Only a few papers have investigated circulating miRNAs in CRC patients with a specific stage or treatment in a search for predictive circulating miRNAs. 

In a paper from 2014, serum expression of miRNAs using a microarray platform was tested. Patients with stage IV CRC and recurrent disease after adjuvant treatment were included. Resistance to treatment with FOLFOX was tested in 16 patients (eight responders *vs.* eight non-responders). Five miRNAs (miR-19a, miR-122, miR-144, miR-221, and miR-222) were tested in a validation group of 72 patients (36 responders *vs.* 36 non-responders). MiR-19a was the only significant miRNA up regulated in the non-responder group. The patients in the non-responder group were also divided into patients with intrinsic resistance and acquired resistance. No difference in miR-19a was seen in these non-responding subgroups [26].

In a large study, Zhang *et al.* analyzed serum miRNAs with the aim of identifying predictive circulating miRNAs for chemotherapy. Forty patients with stages III–IV were grouped into 20 responders and 20 non-responders. The treatment regimen was oxaliplatin based. In a screening, testing, and validation phase, a risk score analysis based on a final panel of five serum miRNAs (miR-20a, miR-130, miR-145, miR-216, and miR-372) was associated with patients who did not respond to treatment [27]. 

Kjersem *et al.* investigated plasma miRNAs in patients with mCRC from the NORDIC VII trial. From the patients included in treatment arm A, receiving bolus 5FU and oxaliplatin, pre-treatment plasma levels of 742 miRNAs were analyzed in 24 patients and the most differentially expressed miRNAs were validated in 150 patients from the same arm. High pre-treatment plasma levels of miR-106a, miR-130b, and miR-484 were seen in non-responders and high pre-treatment levels of miR-27b, miR-148a, and miR-326 were all associated with short PFS. High miR-326 was also alone associated with short OS [28]. 

From a study of 68 patients with mCRC treated with capecitabine, oxaliplatin and bevacizumab, plasma miR-126 level was determined before treatment, 3 weeks after treatment start, and at disease progression. A median increase in plasma miR-126 level was seen in non-responders *versus* a median decrease in responders during treatment. In addition, a correlation between tumor size and corresponding change in plasma level of miR-126 was found. However, no difference in baseline plasma levels of miR-126 was seen in the different response groups. Baseline levels of miR-126 above the median were associated with better PFS [29]. 

Our group investigated 742 miRNAs in whole blood from 138 patients with mCRC treated with third-line cetuximab and irinotecan. From pre-treatment samples, high levels of miR-345, miR-143, miR-34*, miR-628-5p, and miR-886-3p and low levels of miR-324-3p were prognostic for OS. Especially miR-345 was prognostic for OS in the multivariable analysis and for PFS in the *KRAS* wild type subgroup, and high levels of miR-345 levels in whole blood were also associated with lack of response to treatment [30]. 

### 2.4. Normalization Methods

Different normalization methods were used throughout the studies. In four of them, the total number of analyzed miRNAs exceeded 100. The choices of normalization methods were: global mean [30], spike in [26], stable miRNAs [28], and normalization using software from Applied Biosystems [27]. For the studies analyzing a small number of miRNAs, miR-16 was used in four studies [17,18,23,31], miR-191 in one study [20], RNU6B in one study [25], and spike in normalization in three studies [21,22,26].

## 3. Discussion

In this review of the literature, we found that the majority of studies on circulating miRNAs aimed at exploring a diagnostic miRNA profile in patients with CRC. Problems with small overlaps between diagnostic signatures in CRC are well known, and discordant results investigating prognostic miRNAs were also seen in the studies included in our review. Discordant results between miRNA signatures are not only seen in patients with CRC but are also found in patients with other types of cancer, and are especially well-established in breast cancer [33]. The only miRNAs that appeared to be prognostic in more than one study were miR-21, miR-155, and miR-200c. Circulating and tissue miR-21 is perhaps one of the most consistent miRNAs investigated in cancer patients. Circulating miR-21 in patients with CRC was not found to be a strong biomarker for prognosis. Although, two studies reported high levels of miR-21 to be a bad prognostic factor for patient outcome, the opposite was seen in a smaller study. Furthermore, two studies investigating miR-21 did not find a significant association with OS. In general, circulating miR-21 is not only reported to be deregulated in patients with different cancer types, but is also reported associated to inflammatory responses [34,35]. This indicates a more versatile role of miR-21 and not just as a tumor-associated miRNA.

Another frequently reported miRNA is miR-141, which has not been reported as tissue specific, cancer type specific, and perhaps not even cancer specific. Increased levels are seen in pregnant women [36] and in association with preeclampsia [37]. Decreased levels are reported in systemic lupus erythematosus [38]. In patients with prostate cancer, Mitchell *et al.* found that miR-141 could differentiate cancer patients from controls [6] and high levels were associated with poor prognosis [39]. In patients with CRC, high circulating levels were prognostic for short OS but when determined in tissue samples, high levels were associated with longer disease-free survival (DFS) measured in tissue from the resected tumor [40]. The same miRNA has been described in different diseases and conflicting results in patients with CRC. This underlines that discordant results are still a hurdle that must be overcome before circulating miRNAs can be implemented as prognostic or predictive markers in a clinical setting.

The targets of miR-141 and miR-21 are numerous. From the miRTarBase [41] over 600 experimentally validated targets for miR-21 and 43 for miR-141 are found. Six targets, *CLOCK*, *PTEN*, *PPARA*, *TGFB2*. *KLF5,* and *ELAVL4*, were found as mutual targets in both miR-141 and miR-21. The biological meaning of these potential targets is nonetheless unclear with regard to circulating miRNAs.

In the literature, both serum and plasma samples have been used as sources for miRNA detection and accepted as equally preferable. Red and white blood cells along with platelets have been reported as major contributors to circulating miRNAs. In addition, the differences in the blood cell-derived miRNAs may overshadow differences between miRNAs in cancer patients and controls [42]. This problem may also exist in studies on prognostic and predictive miRNAs, and could also be one of the reasons for the discordant results. To optimize the preparation of plasma samples, a double step of centrifugation has been proposed to overcome the above-mentioned problem [43]. Alternatively it is possible to reduce the influence of miRNAs from blood cells by not removing the lowest 5 mm of the supernatant when EDTA plasma and serum were aliquoted after the centrifugation of whole blood.

To identify circulating as opposed to blood cell-derived miRNAs, a complete removal of cellular components is needed. To obtain truly cancer-related circulating miRNAs, further investigation regarding the isolations of microvesicle- or exosome-derived miRNAs is needed. This is currently under investigation and will, hopefully, increase the probability for finding a useful cancer-related circulating miRNA-biomarker within circulating miRNAs. However, even if miRNAs are isolated from exosomes or microvesicles, they may not necessarily be tumor related. 

An important step in the steps of miRNA expression analysis is normalization. Normalization has the purpose of removing the majority of non-biological variation and isolate biological variations for further analyses. Global mean normalization is often used when analyzing a large number of miRNAs. For studies analyzing or validating a small number of miRNAs, the use of reference genes as endogenous control or Spike in are common. In this review, miR-16 and miR-191 were used as endogenous controls. Levels of miR-16 along with miR-451 were stable between patients, but sensitive to hemolysis where levels were increased [44]. In addition, several studies are proposing miR-16 to be deregulated in different types of cancer 

It seems unrealistic that a single miRNA can have a stable expression in many different study designs and across different kinds of cancer diseases [12]. Different normalization methods, with the use of unreliable reference genes, may lead to incorrect selection of prognostic and predictive miRNAs and a consensus of normalization method in studies with circulating miRNAs in CRC patients is needed.

To determine if a miRNA profile, or any other biomarker is predictive, there must be at least two groups available for comparison. Most favorably this should be done in a randomized trial where the predictive biomarker is tested in a group of patients, treated with the drug of interest, compared to a group of patients that does not receive the drug [45]. This was not the case in any of the six studies in this review claiming to have a predictive miRNA profile for treatment. It is still possible that these miRNAs are in fact predictive, but a more optimal study design is necessary to define a true predictive biomarker, as proposed by Ballman [45].

## 4. Conclusions

Circulating miRNAs are still to be considered as promising prognostic or predictive biomarkers for patients with CRC. As seen in this literature review discordant results may be due to a suboptimal study design because of heterogeneous groups, stages, number of patients, and sporadic validation. More focus on technical issues, such as standardization of pre-analytical and analytical procedures, are also of great importance if circulating miRNAs in meaningful tests are to be implemented as biomarkers in a clinical setting.

## Figures and Tables

**Table 1:** Summary of circulating miRNAs as prognostic or/and predictive biomarkers in patients with colorectal cancer.

	miRNA	Effect	Sample	Disease Stage of CRC/Treatment	No of Patients	Outcome	Reference
	miR-21	prognostic	Serum	Stage I to IV	12 Discovery/ 182 Validation	High miR-21 levels associated with tumor size, distant metastasis, and poor survival	Toiyama [16]
miR-21		prognostic	Plasma	Resectable	57 Discovery/ No validation	High levels in plasma drawn from mesenteric vein correlated with shorter DFS	Monzo [20]
	miR-21	prognostic	Serum	Stage I to IV	102 Discovery/ No validation	Low levels associated with short OS along with CEA, age and tumor stage	Menéndez [18]
	miR-21, let-7g, miR-31, miR-92a, miR-181b, and miR-203	prognostic	Serum	Localized or metastatic	30 Discovery/ 83 Validation	The expression levels of the six serum miRNAs were correlated to tumor stage	Wang [23]
	miR-26a, miR-124-5p	prognostic	Plasma	Unresectable or resectable	71 Discovery/ No validation	High miR-124-5p and low miR-26a levels correlated with long OS	Jinushi [25]
	miR-92a	prognostic	Serum	Resectable	200 Discovery/ No validation	High levels associated with poor survival	Liu [17]
	miR-141	prognostic	Plasma	Stage IV	102 Discovery/ 156 Validation	High levels were prognostic for short OS	Cheng [22]
	miR-155	prognostic	Serum	Localized or metastatic	146 Discovery/ No validation	High levels correlated with OS and PFS	Lv [24]
	miR-183	prognostic	Plasma	Stage III and IV	118 Discovery/No validation	High levels associated with local and distant recurrence and short DFS & OS	Yuan [32]
	miR-200c	prognostic	Serum	Stage I to IV	12 Discovery/ 182 Validation	Associated with local and distant recurrence. High level was prognostic for OS	Toiyama [21]
	miR-221	prognostic	Plasma	Stage I to IV	103 Discovery/ No validation	High level was prognostic for short OS	Pu [19]
	miR-19a	prognostic/potentially predictive	Serum	Stage IV/1st line FOLFOX	16 Discovery/ 72 Validation	High levels in non-responders	Chen [26]
	miR-20a, miR-130, miR-145, miR-216 and miR-372	prognostic/potentially predictive	Serum	Stage III & IV/Oxilaplatin based chemotherapy	253 Discovery/ No validation	Risk score analysis from all miRNAs predicts chemoresistance.	Zhang [27]
	miR-106a, miR-484, and miR-130b (response), miR-27b, miR-148a, and miR-326 (PFS & OS)	prognostic/potentially predictive	Plasma	Stage IV/Nordic FLOX	24 Discovery/ 150 Validation	High levels in non-responders. High levels associated with PFS. High miR-326 associated with short OS.	Kjersem [28]
	miR-126	prognostic/potentially predictive	Plasma	Stage IV/1st line chemotherapy + bevacizumab	68 Discovery/ No validation	Changes during treatment separated responders *vs.* non-responders	Hansen [29]
	miR-155, miR-200c, and miR-210	prognostic/potentially predictive	Serum	Stage III/Different chemotherapy regimens with or without VEGF/EGFR inhibitors	15 Discovery/ No validation	High levels predicting distant metastasis and chemoresistance	Chen [31]
	miR-345, miR-143, miR-34*, miR-628-5p, miR-886-3p, miR-324-3p	prognostic/potentially predictive	Whole blood	Stage IV/3rd line irinotecan & cetuximab	138 Discovery/ No validation	High levels prognostic for short OS	Schou & Rossi [30]

* Method: microarray. For all other sqRT-PCROS: Overall Survival, PFS: Progression Free Survival, DFS: Disease Free Survival.
